# A new PEDV strain CH/HLJJS/2022 can challenge current detection methods and vaccines

**DOI:** 10.1186/s12985-023-01961-z

**Published:** 2023-01-20

**Authors:** Xin Yao, Wen-Ting Qiao, Yu-Qian Zhang, Wei-Hong Lu, Zhen-Wei Wang, Hui-Xin Li, Jin-Long Li

**Affiliations:** 1 College of Veterinary Medicine, Northeast Agricultural University, Harbin, 150030 People’s Republic of China; 2 Division of Avian Infectious Diseases, State Key Laboratory of Veterinary Biotechnology, Harbin Veterinary Research Institute, Chinese Academy of Agricultural Sciences, Harbin, 150001 People’s Republic of China; 3 Qianyuanhao Biological Co. Ltd., Beijng, 100070 People’s Republic of China; 4 Key Laboratory of the Provincial Education Department of Heilongjiang for Common Animal Disease Prevention and Treatment, Northeast Agricultural University, Harbin, 150030 People’s Republic of China; 5 Heilongjiang Key Laboratory for Laboratory Animals and Comparative Medicine, Northeast Agricultural University, Harbin, 150030 People’s Republic of China

**Keywords:** Porcine epidemic diarrhea virus, CH/HLJJS/2022, Evolutionary analysis, Typing characteristics, Neutralizing epitope mutations

## Abstract

**Background:**

Porcine epidemic diarrhea virus (PEDV) variant strains cause great economic losses to the global swine industry. However, vaccines do not provide sufficient protection against currently circulating strains due to viral mutations. This study traced the molecular characteristics of the most recent isolates in China and aimed to provide a basis for the prevention and treatment of PEDV.

**Methods:**

We obtained samples from a Chinese diarrheal swine farm in 2022. Reverse transcription polymerase chain reaction and immunofluorescence were used to determine the etiology, and the full-length PEDV genome was sequenced. Nucleotide similarity was calculated using MEGA to construct a phylogenetic tree and DNASTAR. Mutant amino acids were aligned using DNAMAN and modeled by SWISS-MODEL, Phyre2 and FirstGlance in JMOL for protein tertiary structure simulation. Additionally, TMHMM was used for protein function prediction.

**Results:**

A PEDV virulent strain CH/HLJJS/2022 was successfully isolated in China. A genome-wide based phylogenetic analysis suggests that it belongs to the GII subtype, and 96.1–98.9% homology existed in the whole genomes of other strains. For the first time, simultaneous mutations of four amino acids were found in the highly conserved membrane (M) and nucleocapsid (N) proteins, as well as eight amino acid mutations that differed from the vast majority of strains in the spike (S) protein. Three of the mutations alter the S-protein spatial structure. In addition, typing markers exist during strain evolution, but isolates are using the fusion of specific amino acids from multiple variant strains to add additional features, as also demonstrated by protein alignments and 3D models of numerous subtype strains.

**Conclusion:**

The newly isolated prevalent strain CH/HLJJS/2022 belonged to the GII subtype, and thirteen mutations different from other strains were found, including mutations in the highly conserved m and N proteins, and in the S1° and COE neutralizing epitopes of the S protein. PEDV is breaking through original cognitions and moving on a more complex path. Surveillance for PEDV now and in the future and improvements derived from mutant strain vaccines are highly warranted.

**Supplementary Information:**

The online version contains supplementary material available at 10.1186/s12985-023-01961-z.

## Introduction

Porcine epidemic diarrhea virus (PEDV) is a kind of envelope virus with 28 kb sense single stranded RNA,belongs to the α Coronavirus spp. [1]. The genome is composed of 5’UTR-replicase polyprotein 1a/b (ORF1a/b)-spike (S)-ORF3-envelope (E)-membrane (M)-nucleocapsid (N)-3’UTR. PEDV is the main cause of global piglet diarrhea. It was first reported in Europe in 1977, spread widely in East Asia after 2011, and spread in the Americas after 2013, causing huge economic losses to the swine industry [2, 3].

As the virus continues to evolve, scholars refer to the strains that are close to CV777 as classical (GI) and those that are distant become epidemic or virulent (GII) [4]. But there is no unified standard for more detailed division, some scholars believe that named according to the clades of the evolutionary tree, such as a, b, c and so on. It has also been reported that GI and GII divided into three groups GIa, GIb, GIc and GIIa, GIIb, GIIc each, or the variants divided into Asian mutant strains geographically, American virulent strains and American indels strains [1, 5, 6].

Protein S is an important protein. It is a type I glycoprotein composed of subunits S1 and S2 subunit of the viral surface trimmer. It is mediated of PEDV by the binding of an expected receptor amino peptide enzyme N and sialic acid [7, 8]. S1 is involved in receptor binding, and S2 is involved in viral membrane and target cell membrane fusion [9]. The reason why the virus escapes from the host immunity after vaccination is that mutation, deficiency, and insertion in the S protein can alter the epitope of the antigen [10]. The M protein is the most abundant membrane glycoprotein of the viral coat, which is located mainly in the coat, and only a part of the amino terminal glycosylation is exposed  to the outer layer and is an important protein for the assembly and budding of the virus particle, and it can induce the interferon production [11]. The N protein binds to viral RNA and plays an important role in the process of viral gene combinations. It can bind to the membrane and promote assembly and replication of new virus bodies and is very important for the induction of cell immunity [12].

In this study, new three-dimensional structure were found in S protein and M protein from the latest isolates in China. Mutations in S1° and COE regions led to changes in antigen epitopes. At the same time, the isolates added mutations on the basis of typing markers, which were jointly repaired by a few strains in other time spaces, providing a hypothesis for the emergence of new genotypes. In short, these results will provide further complement to the detection and evolution of PEDV, which will help further study the prevention and treatment of PEDV.

## Materials and methods

### Specimen collection and pathogen identification

In 2022, small intestinal tissues were collected from a diarrhoeal pig farm in Heilongjiang Province, China, with clinical symptoms of watery diarrhoea, vomiting, dehydration and rapid weight loss. Intestinal contents of infected piglets from the same house were mixed and sample nucleic acids were extracted using Trizol™ (Thermo, USA) following the manufacturer’s instructions. The gDNA Eraser-treated RNA samples were reverse-transcribed with strand-spe-cific RT primers at 42 °C for 15 min with the PrimeScript® Reverse Transcriptase (Takara, China). Strand-specific quantitative PCR (qPCR) was performed with gene-specific primers and the LightCycler® 480 SYBR Green I Master (Roche, Switzerland) on the QuantStudio™ 5 Real-Time PCR Detection System (Thermo, USA). ORF3 plasmid, flat used as an internal control to normalize gene expression, was kept by the laboratory.

### Cell lines and virus isolation

Cells were maintained in Dulbecco’s modified Eagle’s medium (DMEM, Hyclone, USA) supplemented with 10% fetal bovine serum (Hyclone, USA). Small intestines and their contents, which tested positive by RT-PCR, were homogenized and made into 20% suspensions using DMEM and 100 U/mL penicillin-streptomycin (Hyclone, USA) at 4 °C for 3000 × g for ten minutes, 8000 × g for one minute after aspirating the supernatant. The supernatant was collected and passed through a 0.22 μm filter (Millipore, Billerica, MA, USA) and stored as virus adsorbate at − 80 °C freezer after filtration. When Vero E6 cells (Harbin Veterinary Research Institute, Chinese Academy of Agricultural Sciences, China) were grown to 80% confluence in T25 (Corning, USA) flasks, they were rinsed twice using PBS, inoculated with 2 mL of adsorbate and supplemented with 5 ug/mL pancreatin (Hyclone, USA). After incubation at 37 °C for 2 h, the adsorbent solution was discarded and the cells were rinsed twice using PBS and incubated in 5 mL DMEM supplemented with 2% serum and 5ug/mL pancreatin at 37 °C in 5% CO_2_ for 72 to 84 h. Cultures were placed at − 80 °C for repeated freeze thawing three times, and the mixture was mixed using 0.22 μm filter after marking P1 passage, blind passage was performed after positive RT-qPCR test, and cytopathological effects were observed after the tenth passage.

### Construction of ORF3 plasmids

ORF3 primers were designed based on the sequence of CV777 (AF353511.1) published at National Center for Biotechnology Information (NCBI), the RNA of PEDV CV777 (Harbin Pharmaceutical Group Holding, China) was subjected to PCR using ORF3 F/R, the PCR products were recovered (TIANGEN, China) and ligated to T-Vector pMD19 (Simple, Takara, China) according to the manufacturer’s instructions, and the plasmids from cultured single colonies were extracted and Sanger sequenced (Tsingke Biotechnology, China), and the sequencing results were consistent with the database.

### One step growth curve was plotted

And the one-step growth curve of PEDV was determined with viral titers expressed as 50% tissue culture infectious dose (TCID_50_). Vero E6 cells (2 × 10^6^/mL) were seeded into 6-well cell culture plates and incubated in a 5% CO_2_ incubator for 24 h. Vero E6 cells were then inoculated with PEDV at a multiplicity of infection (MOI) of 1.0 for time point cultures separately up to 72 h. Co-culture for 24 h was selected as the experimental group, and cells cultured in DMEM were used as the control group.

### Indirect immunofluorescence assay (IFA)

Vero E6 cells in six well plates (Corning, China) at 80% confluence were infected with PEDV CV777 and CH/HLJJS/2022 (ON968723.1) for 24 h and then fixed using 4% paraformaldehyde for 30 min. After washing the cells three times using PBS, cells were perforated with 0.2% TritonX-100 (Beyotime, China) for 10 min, and after washing three times, blocking was performed by incubation with 0.3% Bovine Serum Albumin Fraction V (BSA, Sigma, USA) at 37 °C for 30 min. Washed three times with PBS and incubated with mouse anti PEDV N protein monoclonal antibody (Medgene Labs, SD-2–5, USA) for 1 h at 37 °C. After three washes, Alexa fluor 488 (Beyotime, China) conjugated Goat anti mouse IgG was added, incubated for 30 min at 37 °C in dark conditions, washed three times and cells were viewed using an inverted fluorescence microscope (Leica, Germany).

### Genomic sequencing of PEDV CH/HLJJS/2022

Acquisition of a viral second strand was consistent with the method of pathogen identification before library preparation using Nextera XT reagents (Illumina) and sequencing on the NovaSeq 6000 (Illumina, USA) at the Shanghai Tanpu Biotechnology Co., Ltd (Tpbio, China). To remove sequencing adaptors and low-quality reads, raw data were filtered and trimmed by Fastp (v0.20.0). Alignment of the obtained sequencing data was performed with BBmap (v38.51) to the NCBI NT database to remove corresponding rRNA, host and bacterial sequences. De novo genome assembly was performed using SPAdes (v3.14.1) and SOAPdenovo (v2.04). These extracted assembled reads limited the minimum contig length to 100 bases with the best BLAST hits to the NCBI NT database.

### Sequence analysis

Multiple protein amino acid sequences of the reference strain (Additional file 1: Table S2) and CH/HLJJS/2022 were aligned using DNAMAN (v6.0) software. The neighbor joining (NJ) method of MEGA (v6.0) software was used to establish phylogenetic trees for the whole genome and each protein, and the bootstrap value was set to 1000 replicates. ITOL participated in the process of phylogenetic tree change of strains. Genomic and individual gene nucleotide homologies for the reference strain and CH/HLJJS/2022 were analyzed using the MegAlign program in DNASTAR (v7.1.0.44), and the results were analyzed via OmicShare for Heatmap production.

### Protein 3D structure model and function prediction

Homology modeling of the respective protein tertiary structures was performed using Phyre2 and SWISS-MODEL. At the same time Phyre2 validates the above DNAMAN alignment results for the amino acid sequence of each protein. FirstGlance in JMOL verified the amino acid mutation position and SWISS-MODEL verified the effect of the mutation on the structure. TMHMM (v2.0) was used to predict the transmembrane functional changes of S protein and M protein.

### Statistical analysis

Statistical comparisons were performed using GraphPad Prism (version 8.3) software. Student’s t test was used to analyze the data. A *P* value < 0.05 was considered statistically significant. Error bars represent the standard error (± SE). Fluorescence imaging quantitative analysis was performed using ImageJ (version 1.8). Differential coefficients greater than 0.5 were considered statistically significant in the Heatmap.

## Results

### Replication and cell adaptation of CH/HLJJS/2022

**Fig. 1:**
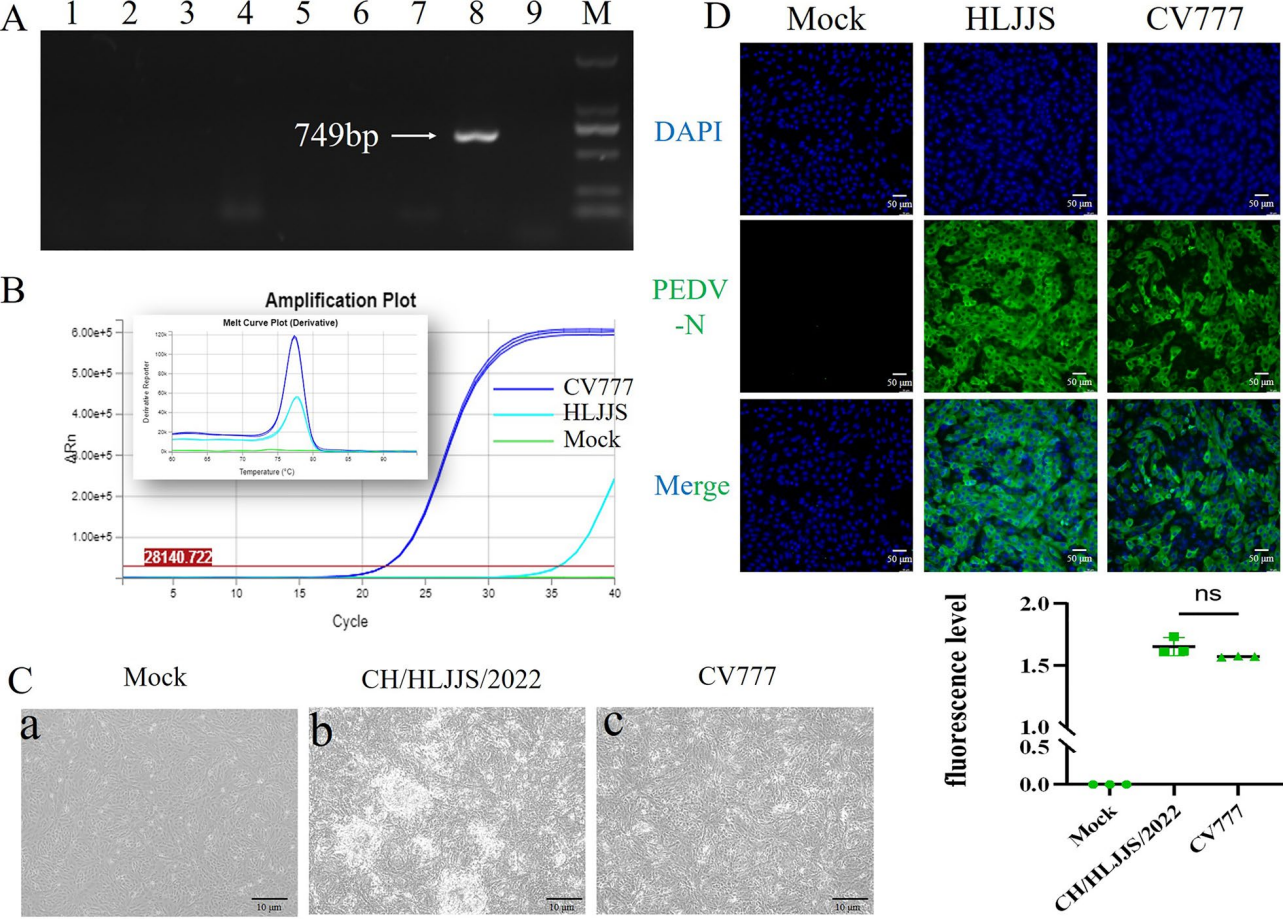
**A** Assays for PCV2, PDCoV, TGEV, PRRSV, PBoV, PRV, PKV, PEDV, BVDV were performed sequentially. **B** The 96 h viral culture fluid, which had been domesticated CV777 as a positive control, was examined. **C** Microscopic observation over 12 generations of blind passage. **D** Immunofluorescence results were obtained at 24 h of infection (n = 3)

**Fig. 2:**
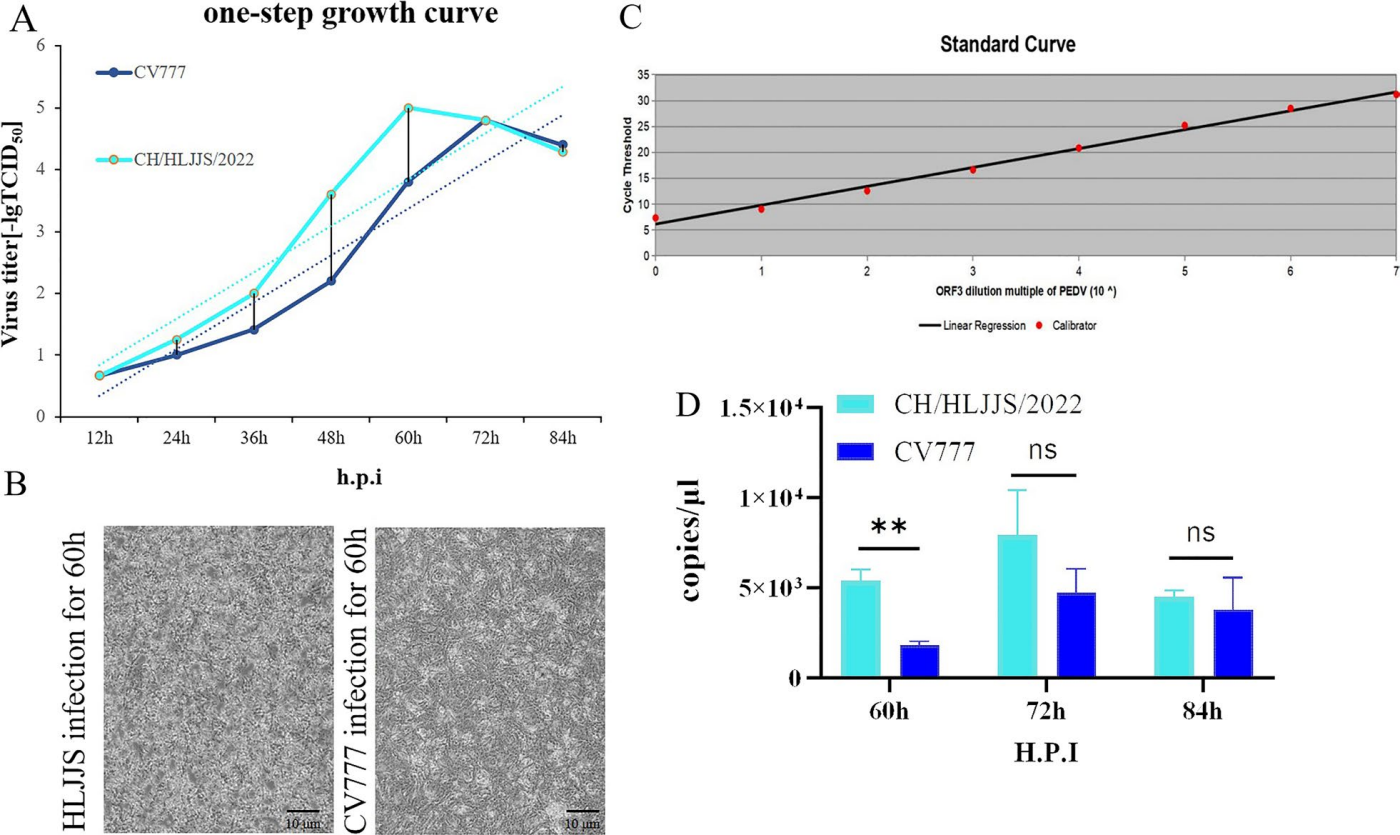
**A** Plot the one-step growth curve of the virus. **B** Infection by the isolated viruses passaged up to passage 12 was observed microscopically for 60 h. **C** ORF3 recombinant plasmids were prepared to generate a standard curve. **D** Viral load assays were performed at 60 h, 72 and 84 h of CH/HLJJS/2022 infection (n = 3, *P* > 0.005)

The total RNA was extracted from the small intestine and its contents in order to determine the diarrhea antigen collected in the pig farm in Heilongjiang province in China, and the RT-PCR result was positive for the PEDV (Fig. 1 A and Additional file 1: Supplementary Table S1). The sample was homogenized to produce an adsorbent, infected with Vero E6 cells, and RT-qPCR was applied to the first venom, with domesticated CV777 used as a positive control (Fig. 1B). CPEs were found upon virus blind passage of the twelfth generation, with the concomitant formation of syncytia as well as cell shedding (Fig. 1C). After 24 h of isolation in Vero E6 cells, IFA showed specific fluorescence with monoclonal antibody against the PEDV N protein, and there was no PEDV in the negative control (Fig. 1D). As a result of the quantitative analysis, CV777 was used as a positive control, and the isolate was adapted. Based on the above results, we named CH/HLJJS/2022. The TCID_50_ was measured by the Reed-Muench method, and a one-step growth curve was drawn, showing that this strain was significantly more virulent than the classical strains at the virulence level (Fig. 2A). It was confirmed by microscopic observation of infection 60 h (Fig. 2B). To better explain this phenomenon, we constructed ORF3 recombinant plasmids targeting conserved sequences of PEDV and plotted a standard curve (Fig. 2C). Viral vector measurements were performed for 60 h, 72 and 84 h of Vero E6 cells, and the results were compatible with the growth curves (Fig. 2D). CH/HLJJS/2022 was more virulent than the classical strains before 72 h, and the virus particles were slowly inactivated and likely degraded in the incubator at 37 °C, possibly due to host death after 72 h.

### Complete genome sequence of CH/HLJJS/2022

**Fig. 3:**
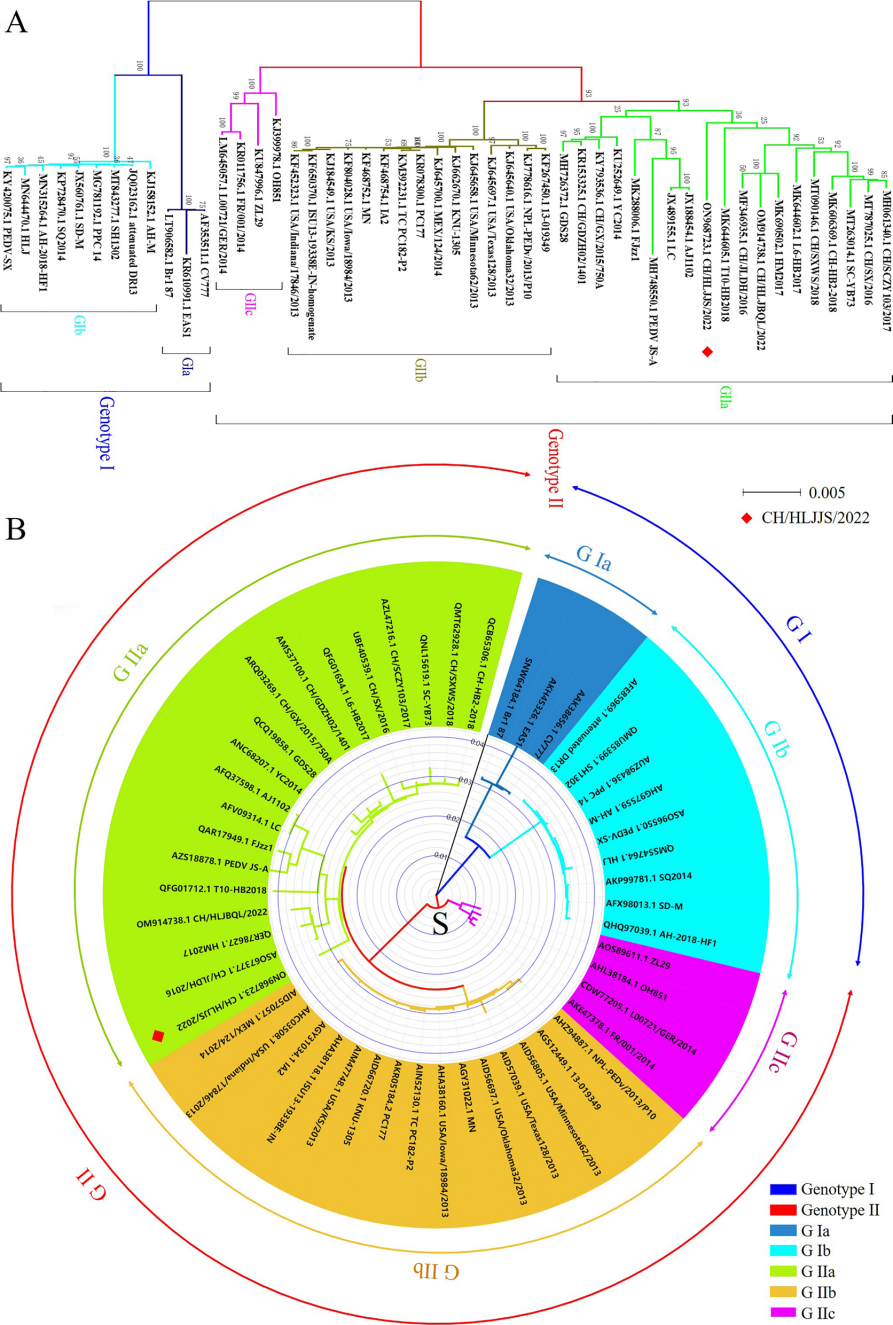
The whole genome and S protein sequences of the CH/HLJJS/2022 strain were analyzed for genetic evolution. **A** The CH/HLJJS/2022 and 49 PEDV strains were subjected to evolutionary analysis and divided into two subtypes, GI and GII, with GIa, GIb and GII further subdivided into GIIa, GIIb, GIIc. **B** S protein is the same as above

**Fig. 4:**
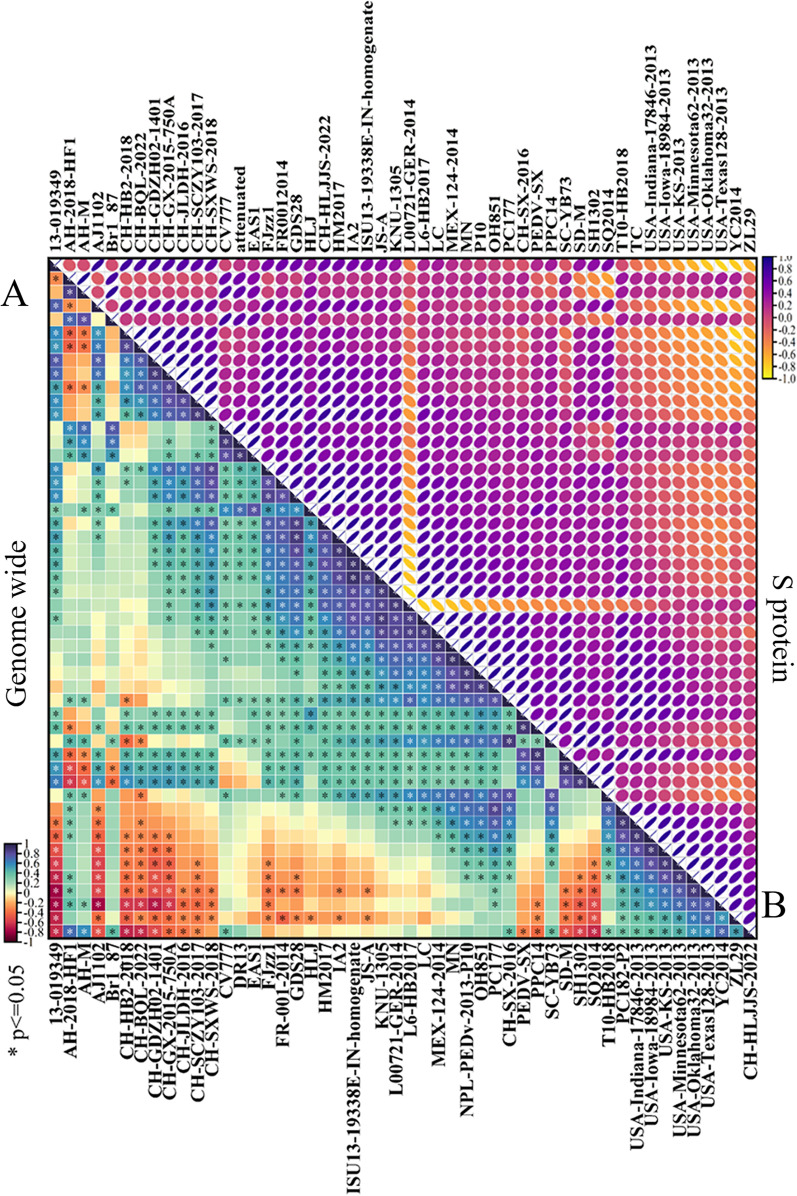
Analysis of the entire genome and homology of the S protein sequence of strain CH/HLJJS/2022. **A** The whole genomes of 50 viruses were analyzed for homology, and the homology results were subjected to a normalized Heatmap. **B** The S protein was processed as above. P ≤ 0.05 were considered statistically significant in the Heatmap

The complete genome sequence of CH/HLJJS/2022 was deduced using Illumina platform and submitted to GenBank with the login number ON968723.1 (Additional file 1: Fig. S1). A total of 28,097 nucleotides were detected for this strain, including ORF1a (nt 300–12,653), ORF1b (nt 12,683–20,644), S (nt 20,641–24,801), ORF3 (nt 24,801–25,475), E (nt 25,456–25,686), M (nt 25,694–26,374), and N (nt 26,386–27,711). The full gene phylogenetic analysis showed that CH/HLJJS/2022 belongs to the GIIa subtype (Fig. 3 A and Additional file 1: Table S2), which is consistent with the developmental analysis of the S gene (Fig. 3B). It has also been maintained at some distance from classical strains in evolutionary analyses of ORF3, E, N, and M proteins, although the N protein is relatively conserved and there are no differences in small protein M (Additional file 1: Fig. S2). Genome wide homology analysis indicated that CH/HLJJS/2022 shared 96.1−98.9% identity with other strains and was most similar to T10-HB2018, while CV777 was shown to share 97.9% nucleotide homology (Additional file 1: Figs. S2 and S3). The homology normalized heat map shows significant differences when compared to the genomes of other strains, and this result was repeated for S and other proteins (Fig. 4 and Additional file 1: Fig. S4).

### Amino acid mutations of CH/HLJJS/2022

**Fig. 5:**
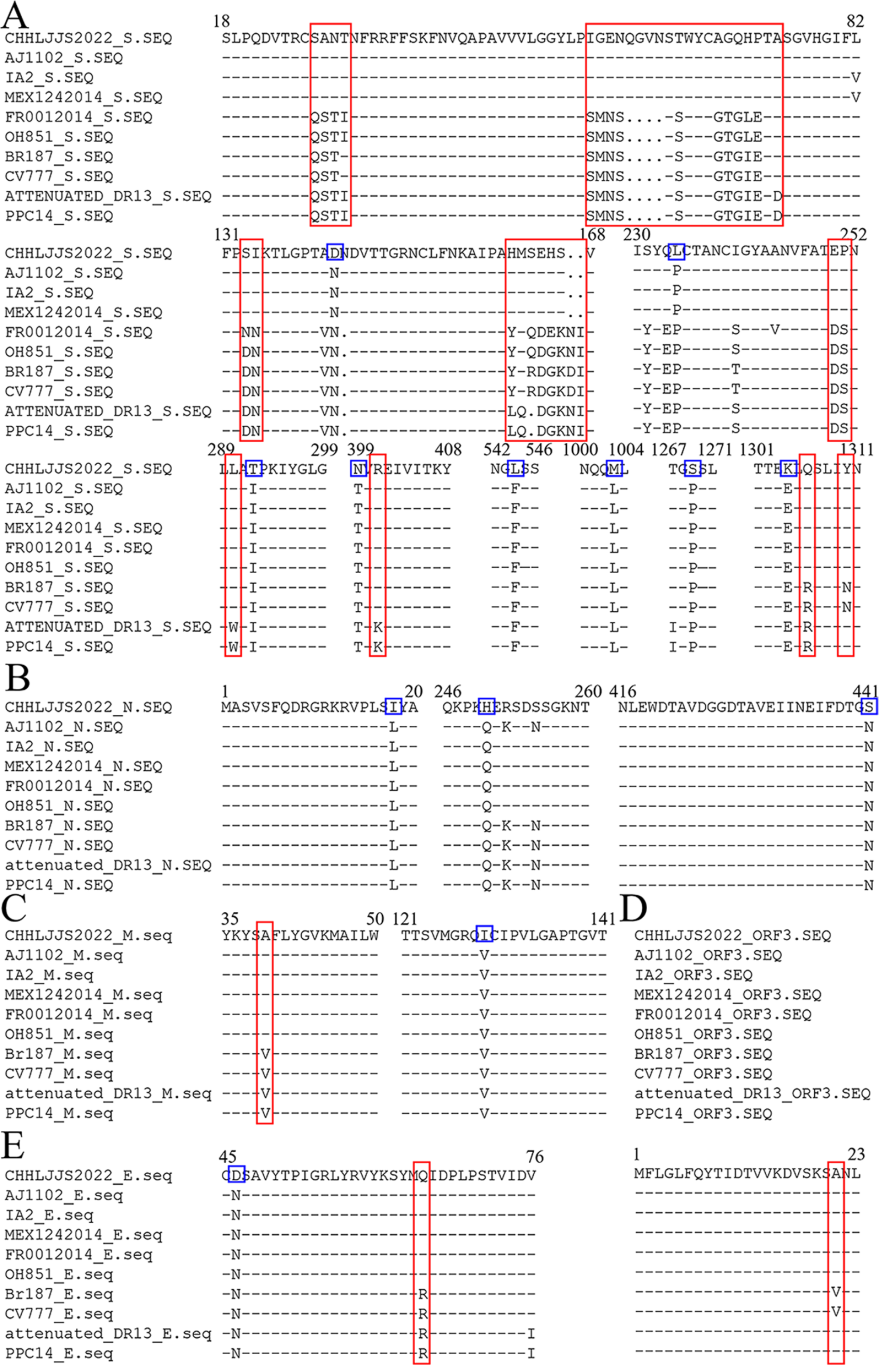
Two strains of each subtype were selected for comparison. **A** S protein to contrast. **B** ORF3 protein to contrast. **C** N protein to contrast. **D** M protein to contrast. **E** E protein to contrast. The red box represents the characteristics of each subtype, and the blue box represents the unique evolutionary characteristics of CH/HLJJS/2022

To determine the specificity of CH/HLJJS/2022, amino acid alignments and analyses were performed using DNAMAN on two randomly selected GIa, GIb, GIIb, and GIIc strains and one additional GIIa strains. The results showed that eight amino acid mutations, N-D (aa 139), P-L (aa 229), I-T (aa 287), T-N (aa 394), F-L (aa 539), L-M (aa 998), P-S (aa 1264) and E-K (aa 1299), were generated located on the S protein (Fig. 5 A and Additional file 1: Fig. S5). Three, one and one amino acid mutations, L-I (aa 18), Q-H (aa 250), N-S (aa 441), V-I (aa 129), and N-D (aa 46) resulted from being located in the N, M and E proteins, respectively (Fig. 5B–E and Additional file 1: Fig. S6).

The results also showed that there was a certain amount of amino acid specific conservation, including S, ORF3, E, and M proteins, among the grouped strains, and these phenomena were the basis for the identification of PEDV grouped strains. Examples are GI/GII group LRS/LQS (aa 1305–1307) in the S protein, GIIa b/c group SANT/QSTI (aa 27–30), GIa/GIb and GII group SVN/SAN (aa 20–22) in the ORF3 protein, GI/GII group MRI/MQI (aa 64–66) in the E protein, GI/GII group SVF/SAF (aa 38–40) in the M protein (Fig. 5). The segregation of CH/HLJJS/2022 implies that novel mutations have arisen in PEDV to include proteins traditionally thought to be highly conserved. While reducing primer accuracy for previous typing assays.

### Mutations of 3-aa altered S protein spatial structure

**Fig. 6:**
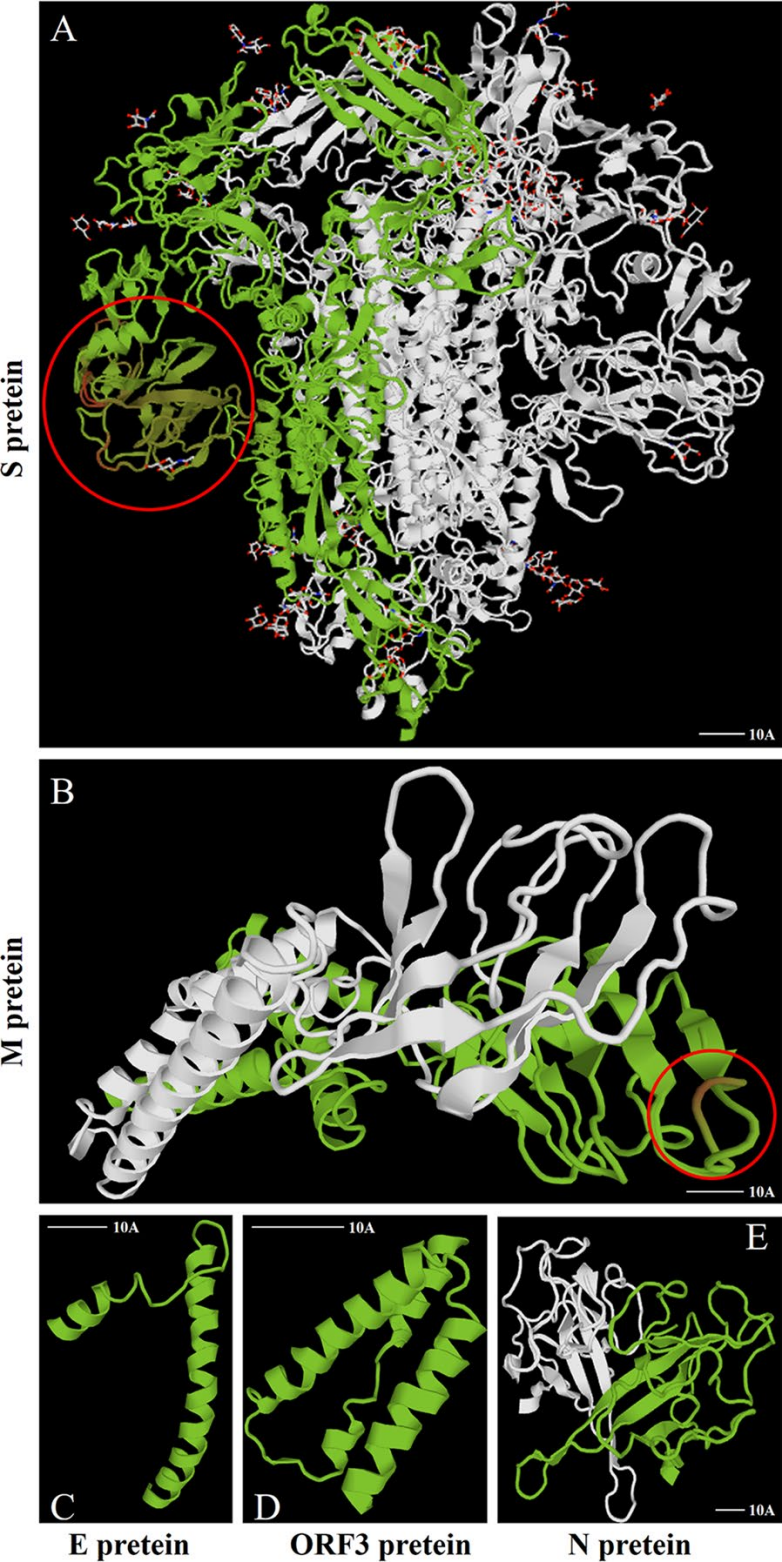
Protein modeling contrasts between CH/HLJJS/2022 and other subtype strains (AJ1102, MEX/124/2014, OH851, CV777 and PPC14). **A** The S protein contrasts with the finding of markedly differential changes in the first 500 amino acids. **B** M protein model. **C** E protein model alignment results. **D** ORF3 protein model. (N) N protein model

**Fig. 7:**
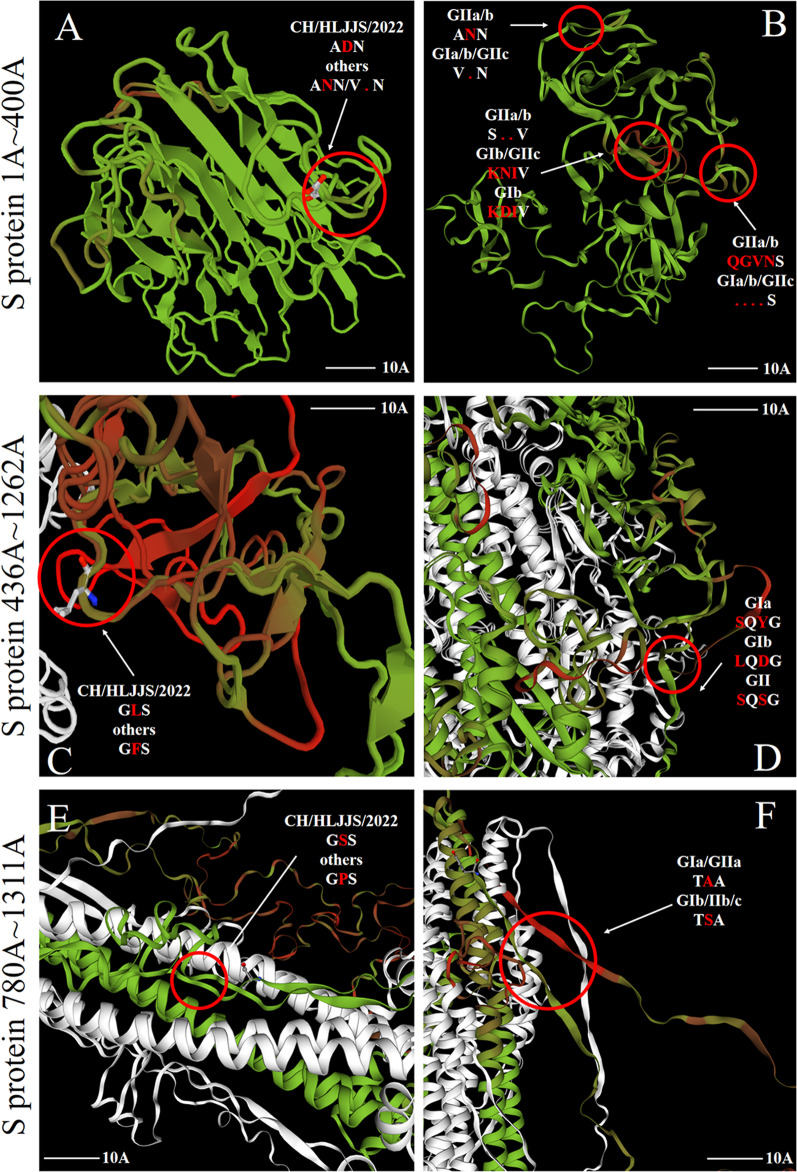
Detailed alignment of the S protein model to the reference strain **A** Structural changes caused by mutation of specific amino acid between CH/HLJJS/2022 strain of S protein and other strains. **B** Structural differences due to grouping landmarks between the various subtypes of S (aa 1–400) protein. **C**–**F** A similar situation

Representative strains from each subtype were selected for protein spatial structure modeling, and structural alterations in the S and M proteins were identified through five sets of subtypes and CH/HLJJS/2022 alignments (Additional file 1: Fig. S7 and Fig. 6). No significant changes were found for ORF3, E and M proteins. To further identify alterations brought about by unique amino acid mutations, we narrowed the simulations to identify specific changes at the mutation site. The results showed that three mutations in the S protein altered the tertiary structure, N-D (aa 139), F-L (aa 539) and P-S (aa 1264) (Fig. 7A, C and E). At the same time, we found that highly conserved amino acids within the subtypes produced divergent structures, consistent with our typing landmark structure derived from our sequence alignment above (Fig. 7B, D and F). To ensure the accuracy of the conclusions, we established the tertiary structure by SWISS-MODEL, which was verified using Phyre2 and FirstGlance in JMOL (Data not shown).

### The 4-aa alters the S antigen epitope

**Fig. 8:**
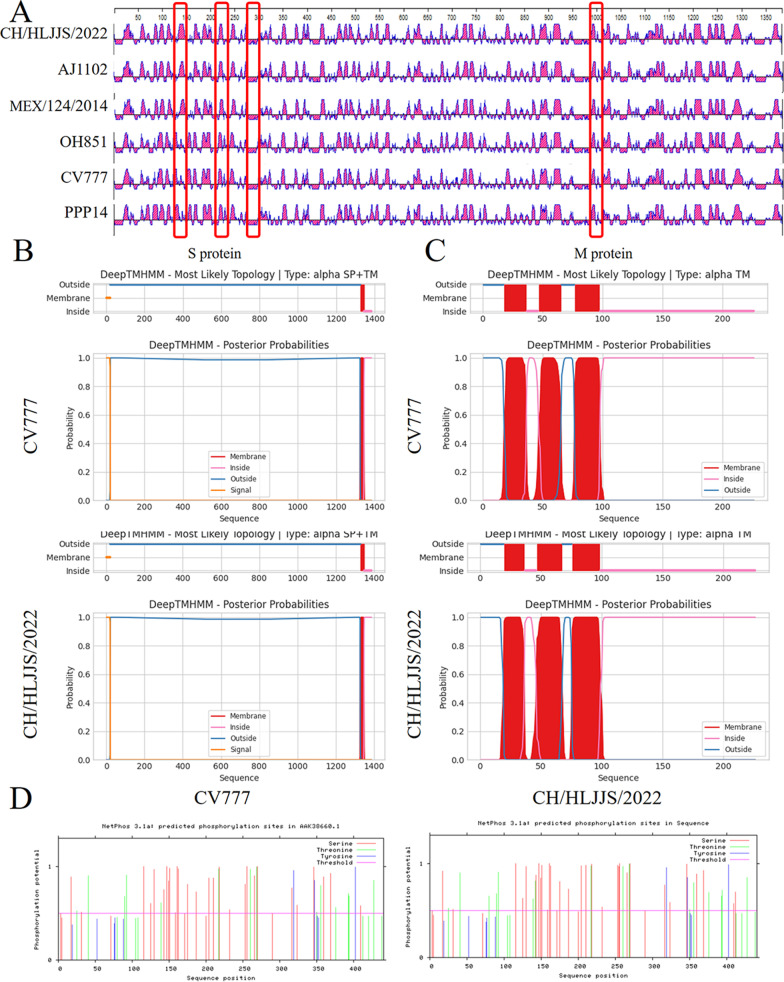
Protein function prediction. **A** Primary epitope comparative analyses of five subtype representative strains and CH/HLJJS/2022 strain revealed 4-aa mutations out finding specific changes. **B** S protein transmembrane function prediction. **C** Prediction of M protein transmembrane function. **D** N protein phosphorylation prediction

Of the eight unique mutations in the S protein found, the S1° and COE neutralizing epitopes were involved. The CH/HLJJS/2022 specific antigenic epitope was found to be distinguished from other strains using DNASTAR, after alignment of the S protein sequence specificity and structural specificity. N-D (aa 139), P-L (aa 229), I-T (aa 287) and L-M (aa 998) showed epitope differences from currently identified PEDV (Fig. 8A).

Although the S protein mutations altered the antigenic epitope compared to CV777, there were no actual changes in transmembrane function (Fig. 8B). The same is true for the transmembrane function prediction of the M protein (Fig. 8C). N-protein S-terminus of CH/HLJJS/2022 allowed enrichment of phosphorylation functions compared to the N-terminus of the other isoforms, but was not revealed due to a prediction score of 0.48 (*P* < 0.5, Fig. 8D).

## Discussion

PEDV is one of the main causes of viral diarrhea, which is a major loss in the world's swine industry. In 1986, China reported the PEDV genome wide CH/S, and since 2011, PEDV has been in fashion in China [13]. It stared in 2013, and it has been discovered for the first time in the United States. As of December 2021, the NCBI reports the whole genome of 811 of all PEDV isolates, the highest of which is 299 isolates from China and 232 isolates from the United States. At the end of the 20th century, PEDV CV777 vaccines were successfully developed, and these inactivated or attenuated vaccines were already widely used in regional pig farms in China and contributed to the early suppression of PEDV infection in China [14]. However, since the newly reported PEDV variant has been overwhelmingly popular since the Chinese and U.S. pandemics in 2011 and 2013, the vaccine derived from classical strains cannot adequately protect the current fashion strain because of the virus mutation [15, 16]. Therefore, field monitoring and analysis of PEDV genes will help to understand the trends of PEDV and help to develop more effective control measures.

The S gene is a commonly used molecular marker in the study of genomic characteristics of PEDV strains. Consists of the S1 antigenic region, the S2 membrane fusion region, and contains four neutralizing epitopes COE (aa 499–638), SS2 (aa 748–755), SS6 (aa 764–771), and 2c10 (aa 1368–1374) [17]. In addition to the four recognized neutralizing epitopes, there are many discovered epitopes such as S1° (aa 1–219), E10E-1–10 (aa 435–485), S1B (aa 510–640), P4B-1 (aa 575–639) and S1D (aa 636–789), among others [17–19]. Mutations in the S protein may alter its antigenic, pathogenic, and neutralizing properties [20]. Detection of amino acid changes in the PEDV S protein therefore helps to understand the evolutionary characteristics of PEDV. In the present study, it was discovered for the first time that the S protein generated 8-aa mutations in the S1°, COE, and aa 229, aa 287, aa 998, aa 1264, aa 1299 regions simultaneously, resulting in protein structural alterations in the S1°, COE and aa 1264 regions of CH/HLJJS/2022 (Fig. 7A, C and E). At the same time, we speculate that the antigenic epitope of CH/HLJJS/2022 appears as a change that distinguishes all subtypes, in two regions of neutralizing epitopes that have been found and one that has not. This may negatively impact the evaluation of vaccine immunity against the above epitopes. In recent years, with the advant of mutant strains, the researchers demonstrated that the existing commercial vaccines (GI) could not provide sufficient immune protection to the epidemic strains (G2), and our findings have demonstrated not only this point of view but alsothe evolving of these strains on a G2. This is a great challenge for our existing G1–G2 junction system.

The immune pressure of vaccine frequently changes the S protein of the virus to maintain the immune escape ability [21]. The M protein plays an important role in the assembly and budding of viral particles, and the M protein is the vaccine candidate antigen because of its ability to production interferon [22]. Recent studies have shown that seven new neutralizing epitopes have been found on the M protein and that it may be possible to produce a vaccine, but unfortunately, the M protein, which has always been considered to be relatively conservative in the CH/HLJJS/2022, has been found [23]. The accuracy of the first technique for establishing molecular biological diagnostics using the N protein is the same as in the case of the M protein (Fig. 5).

The S protein is the major virulence associated protein, and insertions and deletions in the S1 gene result in structural changes on the surface of the protein, with S58_S58insQGVN-N135dup-D158_I159del common pattern mutation (97.28%, 143/147,) [24]. Both GI and GII groups had intergroup concordance for the S protein, with a range of deletions and mutations observed in the GII group compared to classical vaccine strains [25, 26]. These results provide a possibility for our proposed typing marker. The latest studies suggest that vaccines derived from highly virulent PEDV may cross protect against low virulence PEDV infections, and the establishment of an immune barrier using typing markers to screen for virulent strains may become an effective approach in the context of frequent mutations in neutralizing epitopes in circulating strains [27].

In this study, PEDV strains were identified from Chinese pig farms in Mar. 2022 and classified into GIIa subgroup. Compared with classical strains, CH/HLJJS/2022 is unique in sequence with many variations in neutralizing epitopes, suggesting that the development of a new vaccine based on these novel PEDV variants may be necessary to control the PEDV epidemic in China. In addition, in this study, changes of relatively conservative proteins are a major challenge to the original detection methods and candidate vaccine development.

## Conclusion

The present study describes the molecular characterization of a CH/HLJJS/2022 strain isolated from diarrheal piglets in Heilongjiang, China, in 2022. Novel mutations occurred in the S1° and COE regions, and three possible neutralizing epitopes were found, CH/HLJJS/2022 compared with the original classical and epidemic strains. Show that current vaccine immune potency derived from classical and circulating strains is challenged and, in addition, additional alterations in typing markers compared with other subtyped strains create barriers to older means of detection. All the facts indicate that PEDV is acquiring mutations that obsolete detection methods and current vaccines. Our results provide valuable information for the prevention and treatment of PEDV, and will help to further study the evolutionary law.

## Supplementary Information

**Additional file 1**. Supplementary tables and figures.

## Data Availability

The CH/HLJJS/2022 and other PEDV sequences used in this study are available in GenBank of the National Center for Biotechnological Information. The accession numbers of all sequences are showed in Additional file 1: Table S2.
