# Supplementary material for: A new PEDV strain CH/HLJJS/2022 can challenge current detection methods and vaccines
Source: Virol J. 2023 Jan 20;20:13. doi: 10.1186/s12985-023-01961-z (PMC9859669; doi:10.1186/s12985-023-01961-z)
Supplement: Supplementary file 1 — Additional file 1. Supplementary tables and figures. [file 12985_2023_1961_MOESM1_ESM.docx]

***Supplementary Material***

**A new PEDV strain CH/HLJJS/2022 can challenge current detection methods and vaccines**

**Xin Yao^a^, Wen-Ting Qiao^a^, Yu-Qian Zhang^a^, Wei-Hong Lu^a^, Zhen-Wei Wang^c^, Hui-Xin Li^b,^*, Jin-Long Li^a,d,e,^***

^a^ College of Veterinary Medicine, Northeast Agricultural University, Harbin, 150030, PR China

^b^ Division of Avian Infectious Diseases, State Key Laboratory of Veterinary Biotechnology, Harbin Veterinary Research Institute, Chinese Academy of Agricultural Sciences, Harbin 150001, PR China

^c^ Qianyuanhao Biological Co. Ltd. Beijng, 100070, PR China

^d^ Key Laboratory of the Provincial Education Department of Heilongjiang for Common Animal Disease Prevention and Treatment, Northeast Agricultural University, Harbin, 150030, PR China

^e^ Heilongjiang Key Laboratory for Laboratory Animals and Comparative Medicine, Northeast Agricultural University, Harbin, 150030, PR China

* Corresponding author.

**Hui-Xin Li**

Division of Avian Infectious Diseases, State Key Laboratory of Veterinary Biotechnology, Harbin Veterinary Research Institute, Chinese Academy of Agricultural Sciences, Harbin 150001, PR China. E-mail address: lihuixin@caas.cn (H.-X. Li).

**Jin-Long Li**

College of Veterinary Medicine, Northeast Agricultural University, Harbin, 150030, PR China. E-mail address: Jinlongli@neau.edu.cn (J.-L. Li).

**Supplementary Table 1.** The primer sequences.

| Type of Virus | Name of Primer | Sequence of 5’-3’ | Target gene | Fragment length |
| --- | --- | --- | --- | --- |
| PEDV | S1-F | TACCTCCTACTGTCAGGGAAATTGTCA | S | 749bp |
|  | S1-R | GTCTGTGATACCTTCAAGTGGTTTAGG |  |  |
|  | ORF3-F | ATGTTTCTTGGACTTTTTC | ORF3 | 675bp |
|  | ORF3-R | TCATTCACTAATTGTAGCATAC |  |  |
|  | q-F | GCACTTATTGGCAGGCTTTGT | ORF3 | 100bp |
|  | q-R | CCATTGAGAAAAGAAAGTGTCGTAG |  |  |
| PCV2 | ORF2-F | CGGATATTGTAGTCCTGGTCG | ORF2 | 481bp |
|  | ORF2-R | ACTGTCAAGGCTACCACAGTC |  |  |
| PDCoV | N-F | ATGGCTACTGGCTGCGTTAC | N | 383bp |
|  | N-R | GCGTTTCCTGGGCTGATT |  |  |
| TGEV | S2-F | GTGGTTTTGGTYRTAAATGC | S | 859bp |
|  | S2-R | CACTAACCAACGTGGARCTA |  |  |
| PRRSV | Nsp2-F | ATGTTGTGCTTCCTGGGGTTG | Nsp2 | 600-1k bp |
|  | Nsp2-R | CTTGACAGGGAGCTGCTTGA |  |  |
| PBoV | NS1-F | ACAGGCAGCCGATCACTCACTAT | NS1 | 680bp |
|  | NS1-R | CTCGTTCCTCCCATCAGACACTT |  |  |
| PRV | gD-F | GGTGGACCGGCTGCTGAACGA | gD | 455bp |
|  | gD-R | GCTGCTGGTAGAACGGCGTCA |  |  |
| PKV | 3D-F | TGGACGACCAGCTCTTCCTTAAACAC | 3D | 443bp |
|  | 3D-R | AGTGCAAGTGCAAGTCTGGGTTGCAGCCA |  |  |
| BVDV | 5’UTR-F | GGTAGCAACAGTGGTGAG | 5’UTR | 220bp |
|  | 5’UTR-R | GTAGCAATACAGTGGGCC |  |  |

**Supplementary Table 2.** PEDV strains used in this study.

| Accession | Isolate | Collection Date | Geo Location | Genotype |
| --- | --- | --- | --- | --- |
| AF353511.1 | CV777 | 1977 | Belgium | GIa |
| LT906582.1 | Br1/87 | 1987 | United Kingdom | GIa |
| KR610991.1 | EAS1 | 2014 | Thailand | GIa |
| JQ023162.1 | attenuated DR13 | 2009 | South Korea | GIb |
| KJ158152.1 | AH-M | 2011 | China | GIb |
| JX560761.1 | SD-M | 2012 | China | GIb |
| MT843277.1 | SH1302 | 2013 | China | GIb |
| MG781192.1 | PPC 14 | 2014 | South Korea | GIb |
| KP728470.1 | SQ2014 | 2014 | China | GIb |
| MN644470.1 | HLJ | 2015 | China | GIb |
| KY420075.1 | SX | 2015 | China | GIb |
| MN315264.1 | AH-2018-HF1 | 2018 | China | GIb |
| JX188454.1 | AJ1102 | 2011 | China | GIIa |
| MK288006.1 | FJzz1 | 2011 | China | GIIa |
| JX489155.1 | LC | 2011 | China | GIIa |
| MH726372.1 | GDS28 | 2012 | China | GIIa |
| MH748550.1 | JS-A | 2012 | China | GIIa |
| KR153325.1 | CH/GDZH02/1401 | 2014 | China | GIIa |
| KU252649.1 | YC2014 | 2014 | China | GIIa |
| KY793536.1 | CH/GX/2015/750A | 2015 | China | GIIa |
| MF346935.1 | CH/JLDH/2016 | 2016 | China | GIIa |
| MK690502.1 | HM2017 | 2016 | China | GIIa |
| MT787025.1 | CH/SX/2016 | 2016 | China | GIIa |
| MH061340.1 | CH/SCZY103/2017 | 2017 | China | GIIa |
| MK644602.1 | L6-HB2017 | 2017 | China | GIIa |
| MK606369.1 | CH-HB2-2018 | 2018 | China | GIIa |
| MT090146.1 | CH/SXWS/2018 | 2018 | China | GIIa |
| MK644605.1 | T10-HB2018 | 2018 | China | GIIa |
| MT263014.1 | SC-YB73 | 2019 | China | GIIa |
| OM914738.1 | CH/HLJBQL/2022 | 2022 | China | GIIa |
| ON96872301 | CH/HLJJS/2022 | 2022 | China | GIIa |
| KF267450.1 | 13-019349 | 2013 | USA | GIIb |
| KF468754.1 | IA2 | 2013 | USA | GIIb |
| KF650370.1 | ISU13-19338E | 2013 | USA | GIIb |
| KJ662670.1 | KNU-1305 | 2013 | South Korea | GIIb |
| KF468752.1 | MN | 2013 | USA | GIIb |
| KJ778616.1 | NPL-PEDv/2013/P10 | 2013 | USA | GIIb |
| KR078300.1 | PC177 | 2013 | USA | GIIb |
| KM392231.1 | TC PC182-P2 | 2013 | USA | GIIb |
| KF452323.1 | USA/Indiana/17846/2013 | 2013 | USA | GIIb |
| KF804028.1 | USA/Iowa/18984/2013 | 2013 | USA | GIIb |
| KJ184549.1 | USA/KS/2013 | 2013 | USA | GIIb |
| KJ645658.1 | USA/Minnesota62/2013 | 2013 | USA | GIIb |
| KJ645640.1 | USA/Oklahoma32/2013 | 2013 | USA | GIIb |
| KJ645697.1 | USA/Texas128/2013 | 2013 | USA | GIIb |
| KJ645700.1 | MEX/124/2014 | 2014 | Mexico | GIIb |
| KR011756.1 | FR/001/2014 | 2014 | France | GIIc |
| LM645057.1 | L00721/GER/2014 | 2014 | Germany | GIIc |
| KJ399978.1 | OH851 | 2014 | USA | GIIc |
| KU847996.1 | ZL29 | 2015 | China | GIIc |


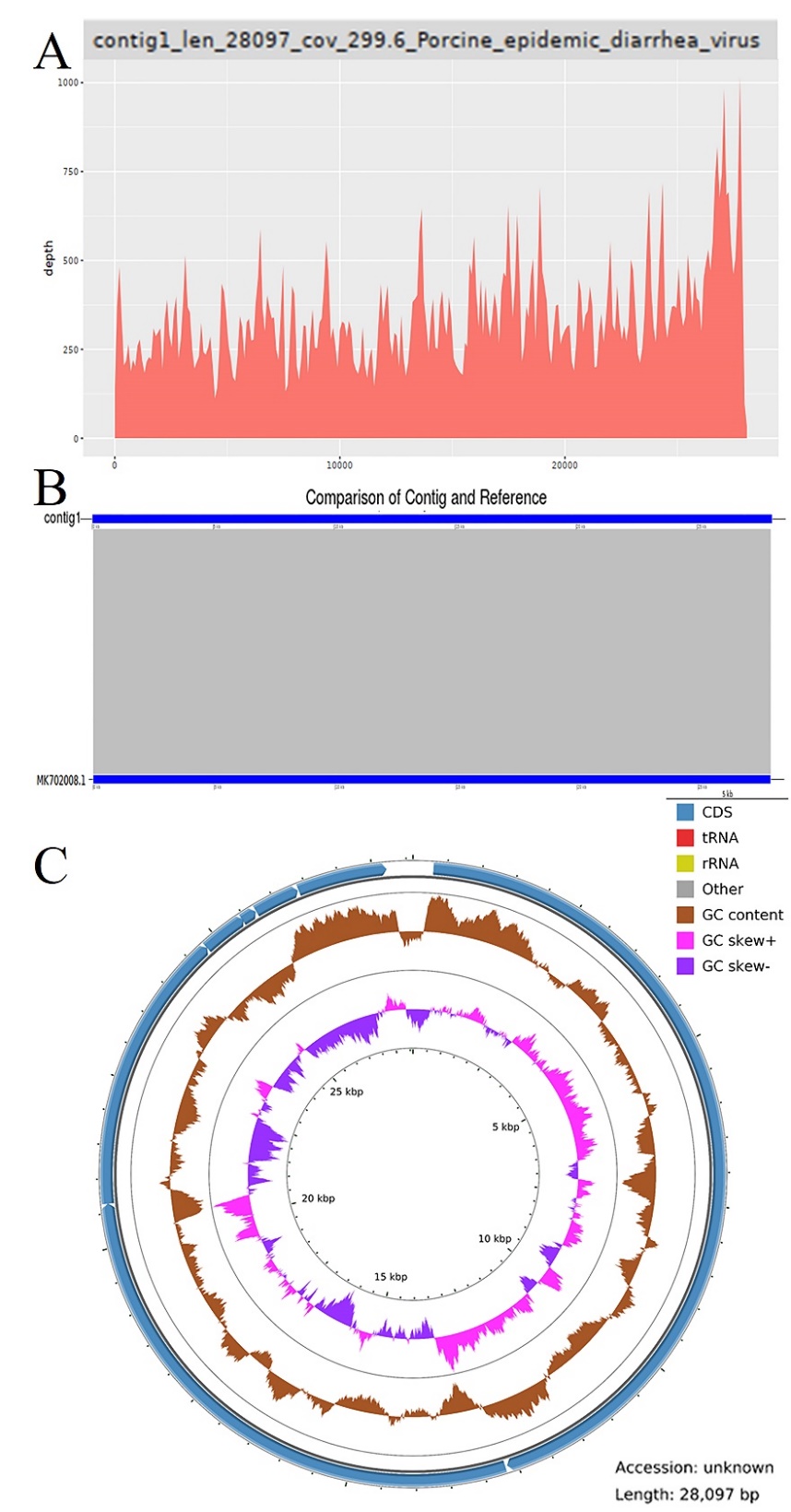


**Supplementary Fig. 1.** Sequencing results of CH/HLJJS/2022. (A) Contigs depth statistical results are presented. (B) Best alignment results display. (C) The assembly result circle diagram exhibits. CDs: CDs fragment after assembled sequence annotation; GC content: display of GC content variation across assembled sequences (sliding windows of varying lengths were selected based on sequence length; contig length < 10000, sliding window length < 50; contig length < 100000, sliding window length 500); GC skew +/-: GC content offset, GC skew = (G - C)/(G + C), which measures the relative content of G and C, gives a positive value for GC skew if G > C and a negative value for G.


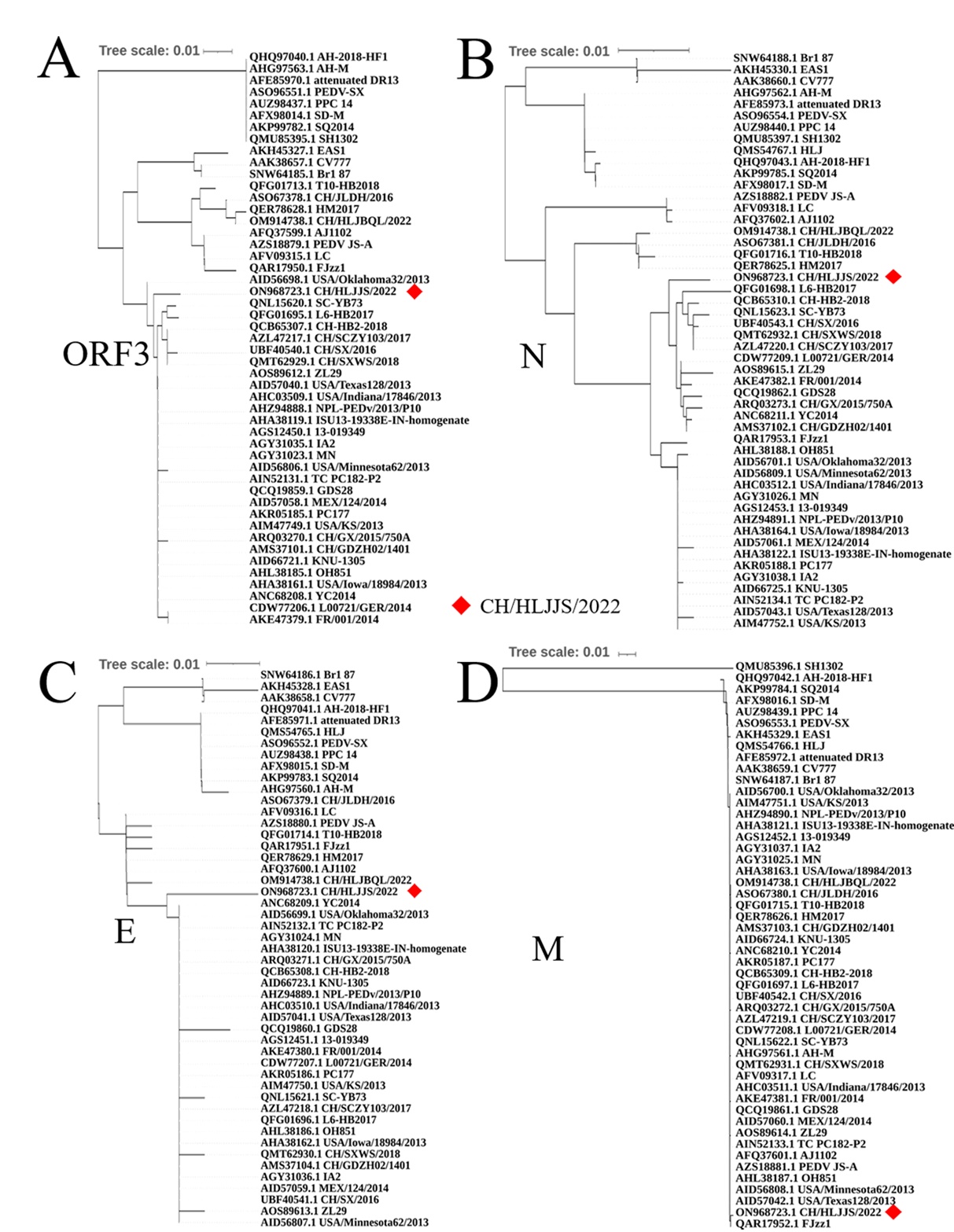


**Supplementary Fig. 2.** Evolutionary analysis of 50 PEDV strains. (A) Evolutionary analysis of ORF3 protein. CH/HLJJS/2022 is marked in red. (B) Evolutionary analysis of N protein. (C) Evolutionary analysis of E protein. (D) Evolutionary analysis of M protein.


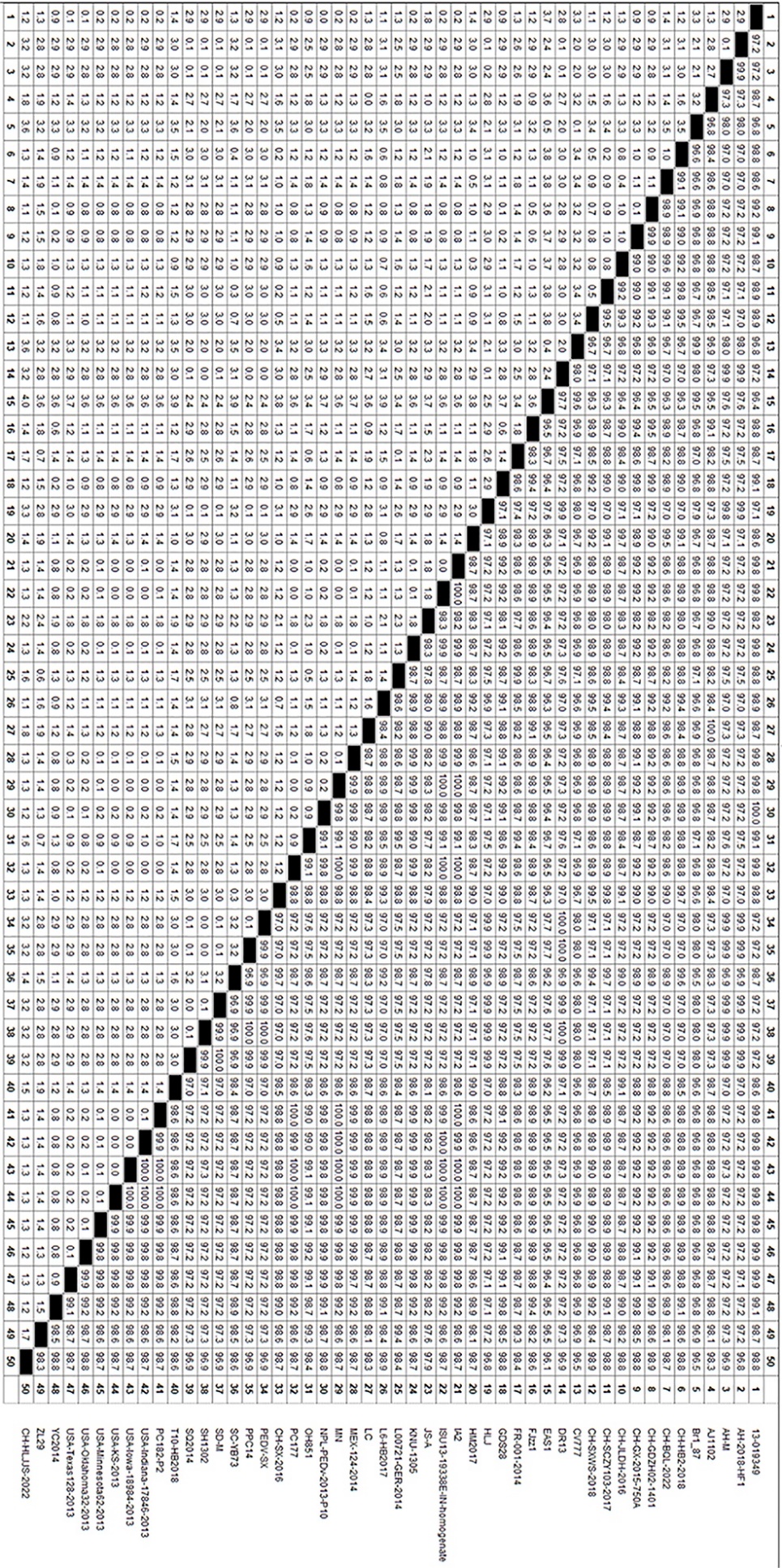


**Supplementary Fig. 3.** Sequence homology analysis of the whole genome of strain CH/HLJJS/2022.


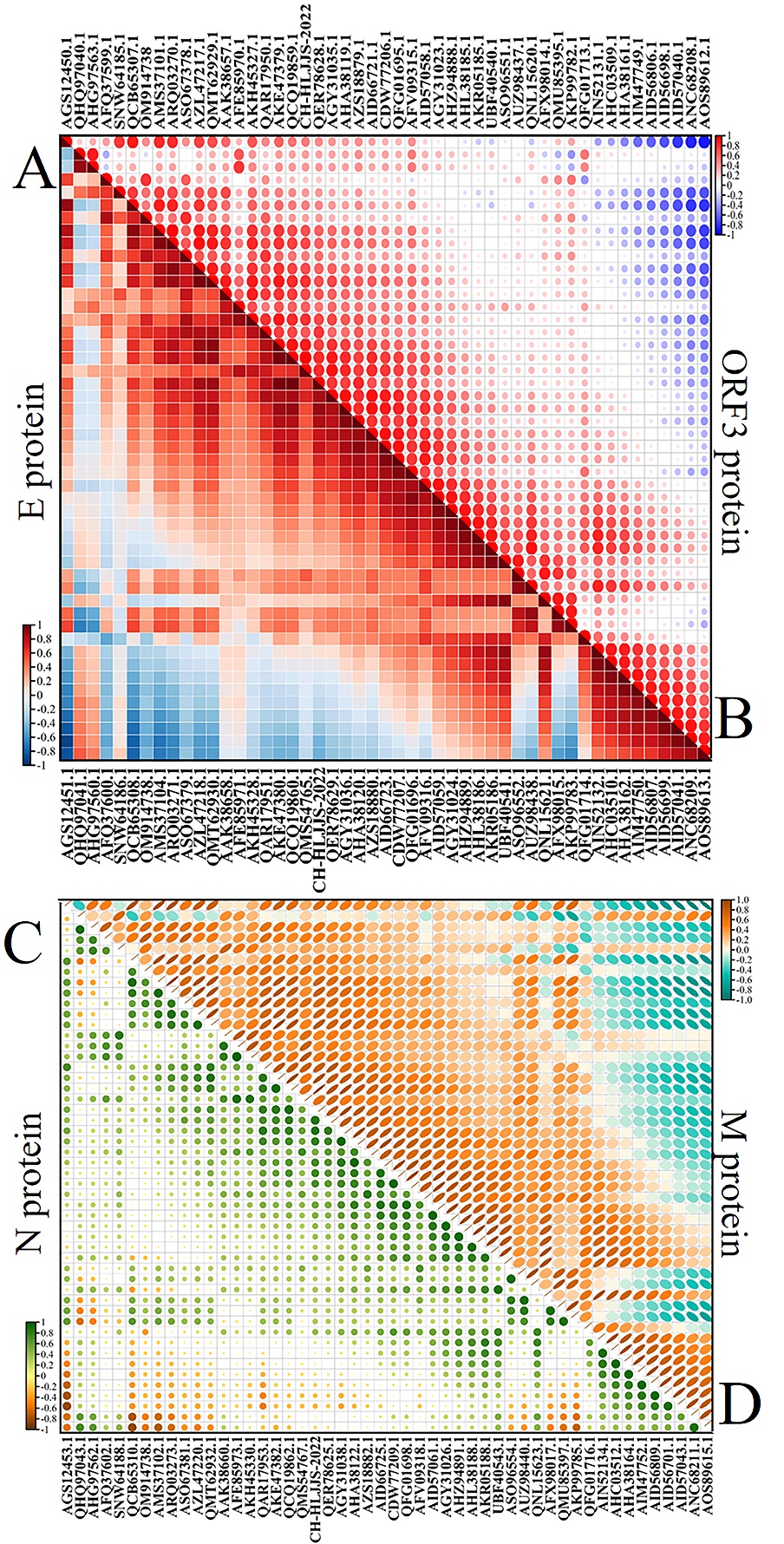


**Supplementary Fig. 4.** The homology of ORF3, E, M and N sequences of strain CH/HLJJS/2022 was analyzed and displayed by heat map normalization. (A) Results of E gene sequence homology thermogram. (B) Results of ORF3 gene sequence homology thermogram. (C) Results of N gene sequence homology thermogram. (D) Results of M gene sequence homology thermogram.


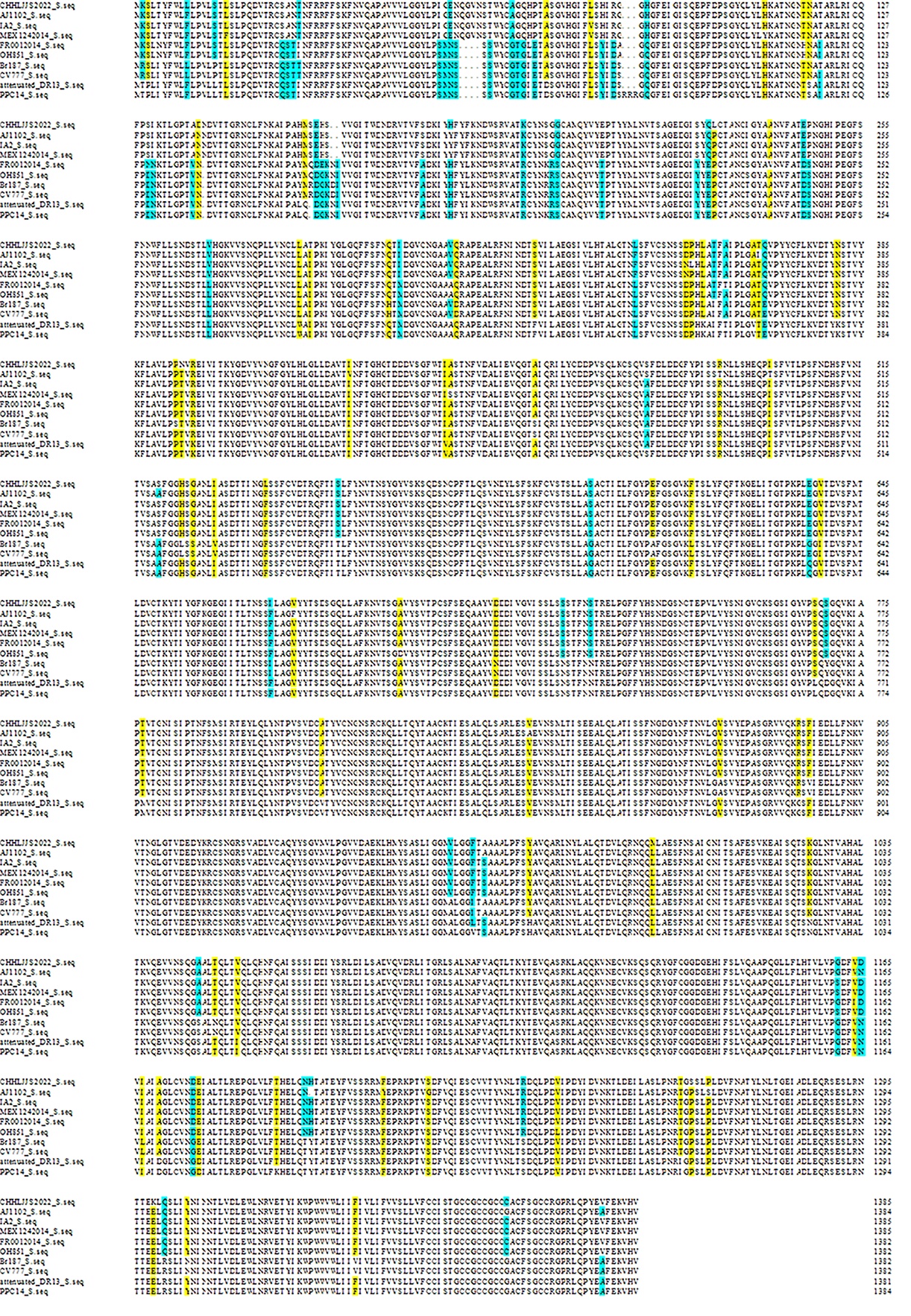


**Supplementary Fig. 5.** 9 representative strains and CH/HLJJS/2022 strain S protein sequence alignment. AJ1102 (JX188454.1), IA2 (KF468754.1), MEX/124/2014 (KJ645700.1), FR/001/2014 (KR011756.1), OH851 (KJ399978.1), Br187 (LT906582.1), CV777 (AF353511.1), attenuated DR13 (JQ023162.1) and PPC 14 (MG781192.1) were aligned with reference.


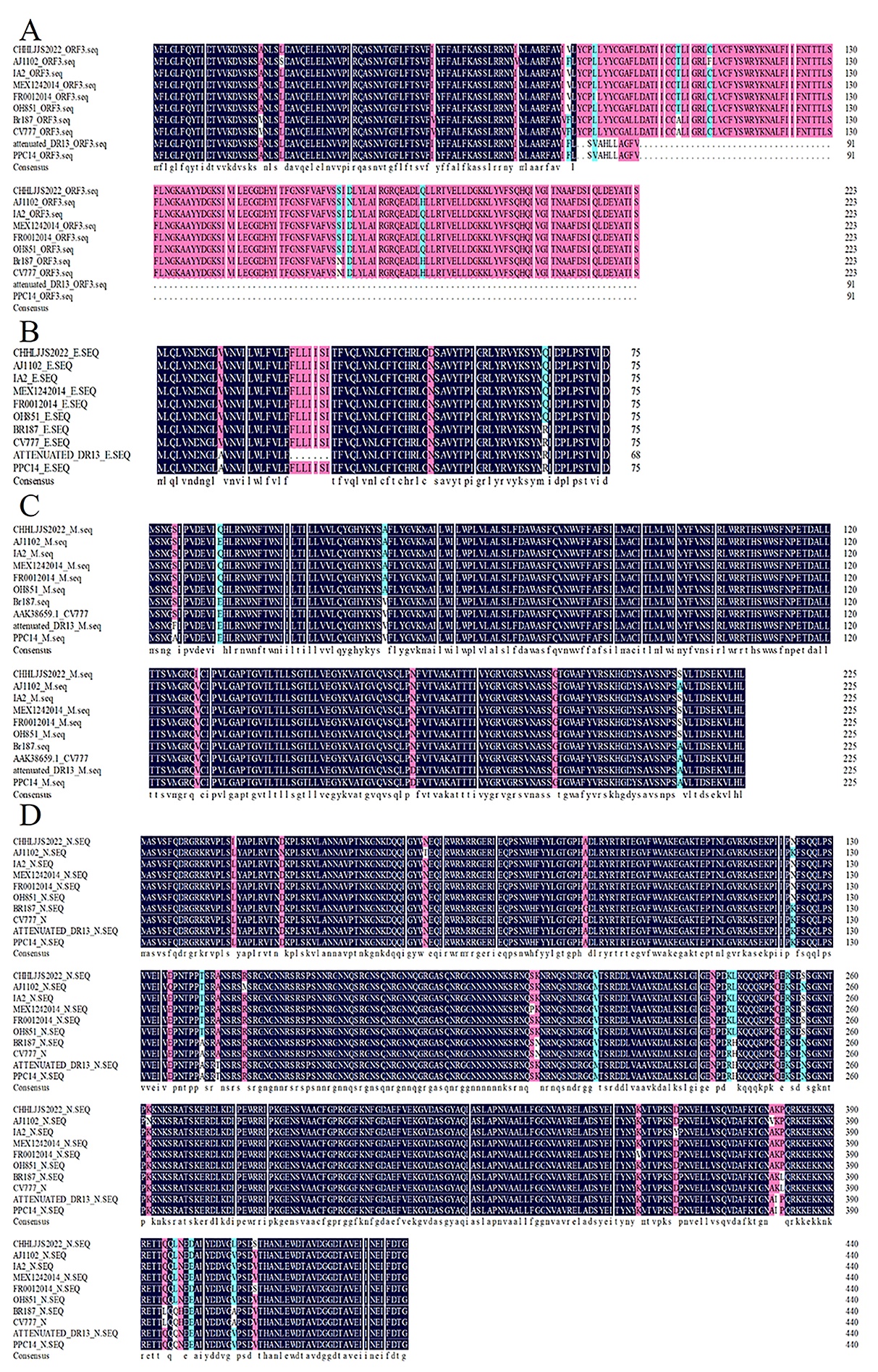


**Supplementary Fig. 6.** 9 representative strains and CH/HLJJS/2022 strain other proteins sequence alignment. (A) Gene nucleotide contrast for ORF3. (B) Gene nucleotide contrast for E. (C) Gene nucleotide contrast for M. (D) Gene nucleotide contrast for N.


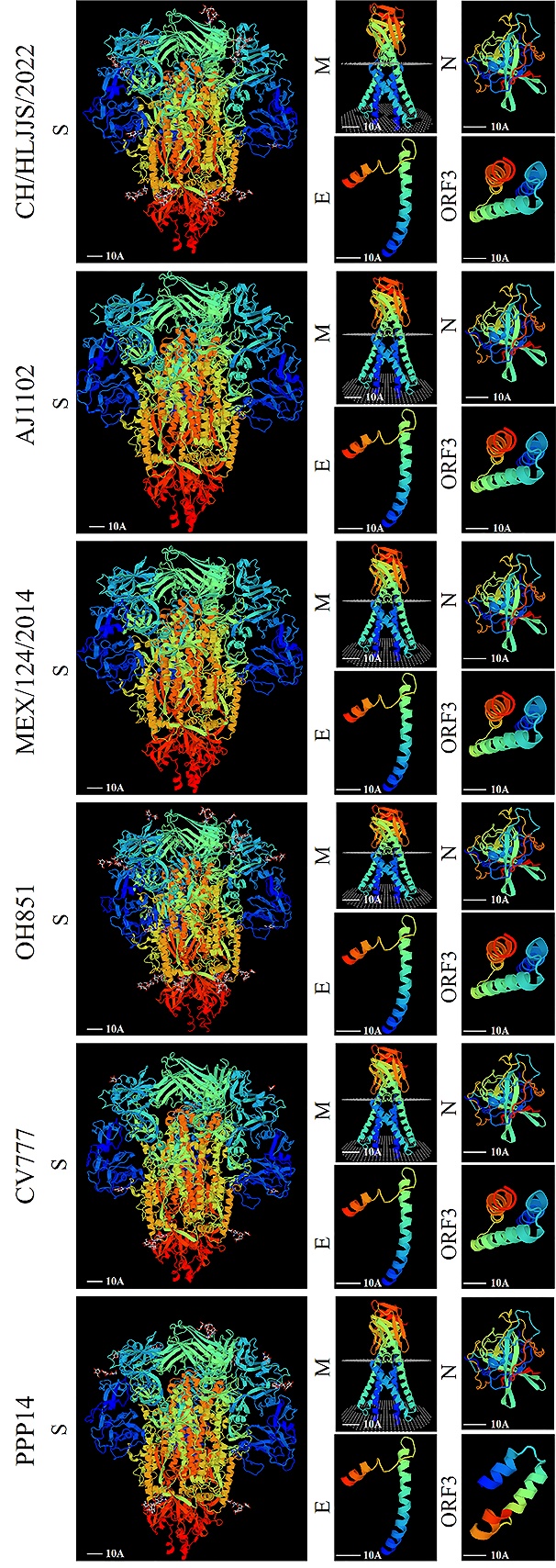


**Supplementary Fig. 7.** Protein tertiary structure modeling was performed on CH/HLJJS/2022 and five representative strains of GIa, GIb, GIIa, GIIb, GIIc. AJ1102, MEX/124/2014, OH851, CV777 and PPC 14 were treated as representative for use.
